# Finite volume analysis of temperature effects induced by active MRI implants with cylindrical symmetry: 1. Properly working devices

**DOI:** 10.1186/1475-925X-4-25

**Published:** 2005-04-08

**Authors:** Martin HJ Busch, Wolfgang Vollmann, Jörg Schnorr, Dietrich HW Grönemeyer

**Affiliations:** 1 Research and Development Center for Microtherapy (EFMT), D-44799 Bochum, Germany; 2 TFH University of Applied Sciences, D-13353 Berlin, Germany; 3 Institut für Radiologie, Charité, Medizinische Fakultät, Humboldt-Universität zu Berlin, D-10117 Berlin, Germany; 4 Grönemeyer Institute for Microtherapy, University of Witten/Herdecke, D-44799 Bochum, Germany

## Abstract

**Background:**

Active Magnetic Resonance Imaging implants are constructed as resonators tuned to the Larmor frequency of a magnetic resonance system with a specific field strength. The resonating circuit may be embedded into or added to the normal metallic implant structure. The resonators build inductively coupled wireless transmit and receive coils and can amplify the signal, normally decreased by eddy currents, inside metallic structures without affecting the rest of the spin ensemble. During magnetic resonance imaging the resonators generate heat, which is additional to the usual one described by the specific absorption rate. This induces temperature increases of the tissue around the circuit paths and inside the lumen of an active implant and may negatively influence patient safety.

**Methods:**

This investigation provides an overview of the supplementary power absorbed by active implants with a cylindrical geometry, corresponding to vessel implants such as stents, stent grafts or vena cava filters. The knowledge of the overall absorbed power is used in a finite volume analysis to estimate temperature maps around different implant structures inside homogeneous tissue under worst-case assumptions. The "worst-case scenario" assumes thermal heat conduction without blood perfusion inside the tissue around the implant and mostly without any cooling due to blood flow inside vessels.

**Results:**

The additional power loss of a resonator is proportional to the volume and the quality factor, as well as the field strength of the MRI system and the specific absorption rate of the applied sequence. For properly working devices the finite volume analysis showed only tolerable heating during MRI investigations in most cases. Only resonators transforming a few hundred mW into heat may reach temperature increases over 5 K. This requires resonators with volumes of several ten cubic centimeters, short inductor circuit paths with only a few 10 cm and a quality factor above ten. Using MR sequences, for which the MRI system manufacturer declares the highest specific absorption rate of 4 W/kg, vascular implants with a realistic construction, size and quality factor do not show temperature increases over a critical value of 5 K.

**Conclusion:**

The results show dangerous heating for the assumed "worst-case scenario" only for constructions not acceptable for vascular implants. Realistic devices are safe with respect to temperature increases. However, this investigation discusses only properly working devices. Ruptures or partial ruptures of the wires carrying the electric current of the resonance circuits or other defects can set up a power source inside an extremely small volume. The temperature maps around such possible "hot spots" should be analyzed in an additional investigation.

## Background

**Figure 1:**
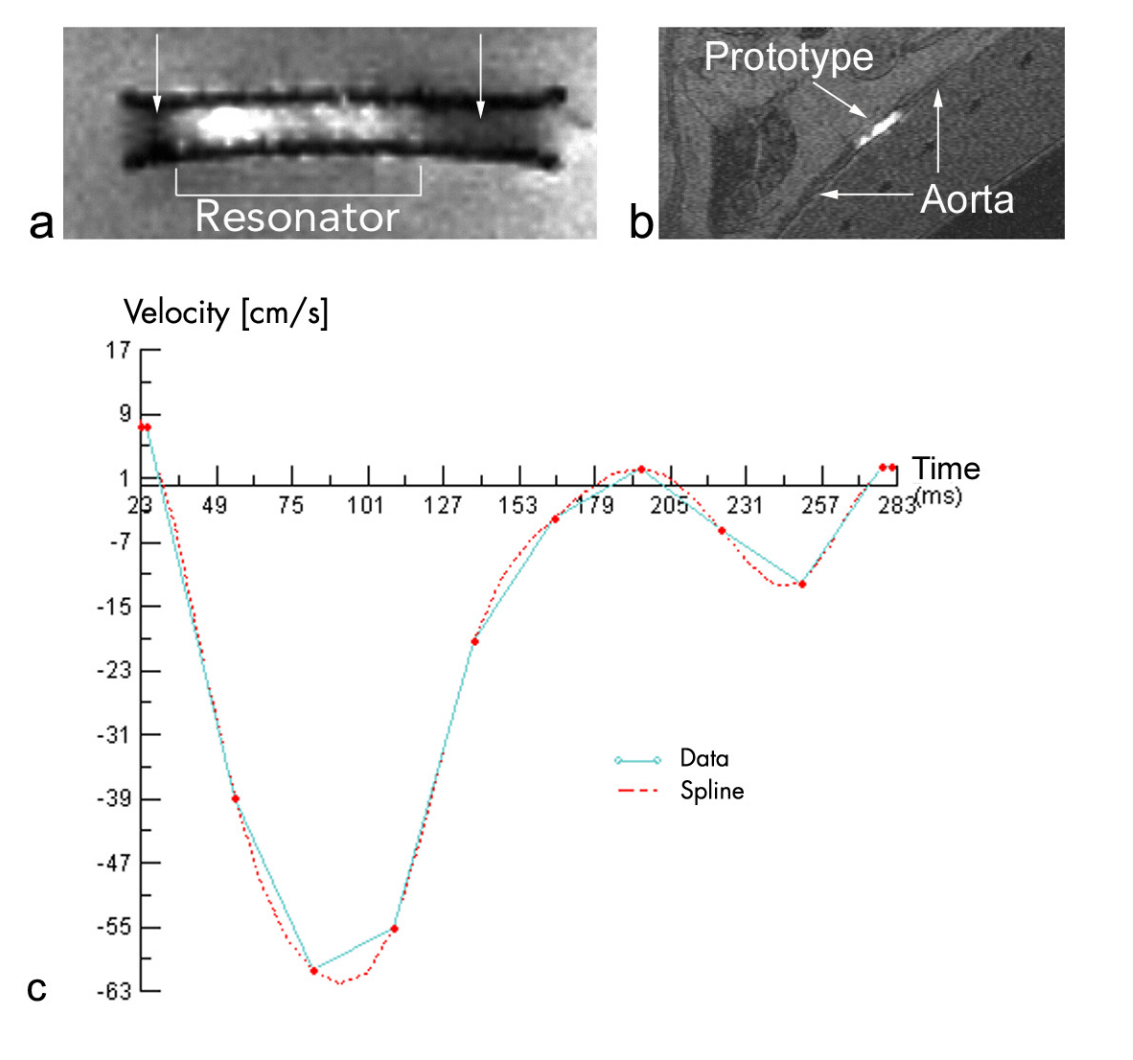
**Commercial Stent with additional resonator inside a water bath (a) and an implanted prototype in a rabbit aorta (b) with velocity curve over the cardiac cycle (c). **a) shows an MR image of a commercially available nitinol stent with an additional resonator around a part of the metallic structure immersed in a water bath. The MR signal inside the stent lumen decreases outside the resonator (arrows) in contrast to the enhanced signal inside the resonator. b) shows one example of an in vivo image with a prototype implanted into a rabbit aorta [9, 10]. c) shows the measured velocity curve inside the prototype of image b and the spline approximation delivered by the the MRI System.

Metallic implants often cause distortions inside Magnetic Resonance (MR) images. These effects arise either from the different susceptibility of tissue and metal, generating a discontinuity of the local field strength at the interface, or from the Faraday cage effect, which is set up by induced eddy currents on the metallic implant structure [1-4]. An advantageous solution to overcome the eddy current shielding is to amplify the transmitted signal to and the detected signal from the spin ensemble appropriately for the Faraday cage. A local amplification of excitation and detection for a magnetic resonance signal can be achieved by using a resonator tuned to the resonance frequency of the MR system [5-8]. Such a resonator can be integrated into the metallic structure of the implant itself or can be added around the normal implant structure. The great advantage of such an active Magnetic Resonance Imaging implant (aMRIi) is the local amplification of the signal only where it is needed. The decreased excitation pulse can be amplified for the spin ensemble inside the faraday cage without affecting the signal and contrast behavior of the rest of the excited volume. The possible advantages of active MRI implants are demonstrated in Figure 1 showing the possible amplification inside a Faraday cage and the potential to get functional information out of such an implant.

The amplification is adjustable by the quality factor *Q *of the resonance circuit and allows the imaging of the inner volume of such metallic implants as well as gathering functional information from this location, for example, velocity or flow. For these special investigations inside the implant volume the quality factor should be as high as possible to increase the signal to noise ratio for this region and to get as low signal as possible from spins outside the volume of interest. For an overall overview of the implant inside the anatomic surrounding it is only necessary to raise the decreased signal inside the Faraday cage to the normal level, which is possible with a lower quality factor. The active implant locally increases the absorbed power during MR investigations. The temperature increase induced by this extra power loss around vessel implants with a cylindrical geometry is calculated with a finite volume analysis for a "worst-case scenario". For most of the simulations only thermal heat conduction is assumed as "worst case" without cooling due to blood perfusion and without cooling due to blood flow inside the active implant. Partly also a blood flow is considered carrying energy out of the simulation volume. The calculations are carried out with respect to power loss, time of rf-exposure and size of the aMRIi. The aim of this investigation was to perform a detailed assessment of the safety for such implants under "worst case" assumptions.

## Methods

### Theory

#### Specific Absorption Rate (SAR)

The local specific absorption rate (SAR; [W/kg]) for electromagnetic radiation is defined as power absorbed per unit of mass of an object at location **r **[11-16],



where *σ *[S/m] is the electric conductivity, *ρ *[kg/m^3^] is the mass density, *E *[V/m] is the amplitude of a sinusoidal time dependent electric field and **r **is the position vector. Assuming that the amplitude of the electric field only arises by induction from a uniform, linearly polarized magnetic field with amplitude *B*_m _[T] and angular frequency *ω *[rad/s] in a homogeneous body of rotational symmetry, the following equation holds [11]



with *r *as component of cylindrical coordinates and *r *= 0 on the rotational symmetry axis aligned parallel to the magnetic field *B*_m_. Combining Eq. (1,2) the spatially averaged SAR over a cylinder with radius *R*_cyl _and using dV = 2 *π *r h dr yields



For pulsed MR sequences two correction factors are necessary,



The duty cycle factor *c*_dc _equals N*τ/TR, where *TR *[s] is the MR-sequence repetition time, *N *is the number of identical excitations during *TR *and *τ *[s] is the duration of one excitation pulse. *c*_dc _equals to the time ratio of "rf-on" to "rf-on + rf-off" during an MR scan. *c*_pwm _corrects for rf-pulses which are not rectangular in shape and is the ratio between the energy of the MR excitation pulse and the energy of a rectangular shaped pulse with identical amplitude and identical duration.

#### Principles of B_1_-field amplification inside a resonator

A circularly polarized magnetic field with magnitude *B*_1 _or a linearly polarized magnetic field with amplitude *B*_m _for excitation is amplified inside an aMRIi with respect to the used resonance circuit. Because of the shape of vascular implants and to simplify calculations, a solenoid is chosen as inductor of the resonator and is investigated as a magnetic antenna. For the theoretical estimation, the axis of the solenoid resonator is assumed to be parallel to a sinusoidal linearly polarized magnetic field with amplitude *B*_m_, or equivalently to be in the plane of a circularly polarized magnetic field with magnitude *B*_1_. For the resonance case (*2πν*_0 _= *ω*_0 _= (*LC*)^-1/2^), where the overall resistance of the resonator is just *R*, the following equation can be derived from the law of induction, Ohms law and the definition of the quality factor Q of a resonator,



where *V*_ind _[V] and *V*_self _[V] are the induced and self-induced voltage respectively, *Z *[Ω] is the impedance, *R *[Ω] is the resistance, *L *[H] is the inductance of the inductor and *α*_res _and *α *are the flip angles inside the resonator inductance and outside the resonator respectively. The total magnetic field inside the resonator arises from both components *B*_m _and *B*_res_. For large quality factors (*Q*>>1) *B*_res _dominates *B*_m_. With Eqs(3, 4) it is possible to calculate the magnetically induced SAR and with the SAR the corresponding power loss inside a resonator. But this takes into account only power losses due to eddy currents and not the total power loss of a resonator.

#### Total power loss of a resonator

The overall power loss P [W] also includes the electric losses on and around the resonator. It is given by the following basic equation [16],



where *W*_total _[J] is the energy stored inside the resonator.

The total energy *W*_total _can be expressed by the energy stored in the magnetic field of maximum amplitude using the following basic relations of a solenoid inductance with n_r _turns (),



where V, A, ℓ with index imp are the volume, cross section and length of the implant inductor. For a pulsed MR sequence with excitation magnitude *B*_1 _the total power loss can be calculated by combining Eqs. (4, 5, 6) to



A comparison of Eqs. (3b, 7) shows that for a certain resonator tuned to the resonance frequency of a specific MR field strength, the supplementary absorbed power is proportional to the SAR of the MR sequence due to the identical dependence on *c*_dc_, *c*_pwm _and B_1_^2^. For a specific sequence on a MRI system with a definite resonance frequency, the extra power is proportional to the quality factor *Q *and the volume *V*_imp _of the inductance (Eq. (7)). The proportionality to the quality factor *Q *may be surprising, because it states that for better quality factors the power loss is larger. This is due to the total energy of the resonator, which depends quadratically on the field strength inside the resonator. This field strength is proportional to the exciting field *B*_1 _(or *B*_m_) as well as the quality factor *Q*. Combining Eq. (5) and Eq. (6) alters the inverse proportionality to *Q *(Eq. (5)) to a proportionality (Eq. (7)) with respect to the field established by the transmit coil. Examining the power loss with respect to the magnetic field inside the resonator confirms the inverse proportionality to *Q*.

### Finite volume analysis

#### Principles

All temperature increases are calculated as temperature difference maps by using a simulation volume divided into many small simulation cells. The energy exchange Δ*E *[J] between two simulation cells with a specific contact area *A *[m^2^], temperature difference Δ*T *[K] and heat diffusion path length Δ*x *[m] for a time interval Δ*t *[s] is given by the equation for heat conduction as,



where *λ *[W/(m K)] is the thermal conductivity.

The total energy change ΔE_tot _of one cell during a time interval Δt is the sum of exchanges of this cell with all adjacent cells with non zero contact area and the energy change due to the power loss p_cell _inside the cell. From this total energy change the temperature difference ΔT* can be calculated,





where *c *[J/(kg K)] is the specific thermal capacity of the cell material and *V*_cell _is the cell volume.

This investigation did not use any commercially available finite volume package. The simulation is self-coded for problems with cylindrical geometry in Kylix and Delphi, a software development environment, based on object oriented Pascal. The graphical outputs are mostly generated by an evaluation (registered *β*-test) version of Teechart 7 used within the Delphi and Kylix environment. The simulation describes the time developing temperature difference maps around an aMRIi with cylindrical geometry for a constant total power loss *P*.

**Figure 2:**
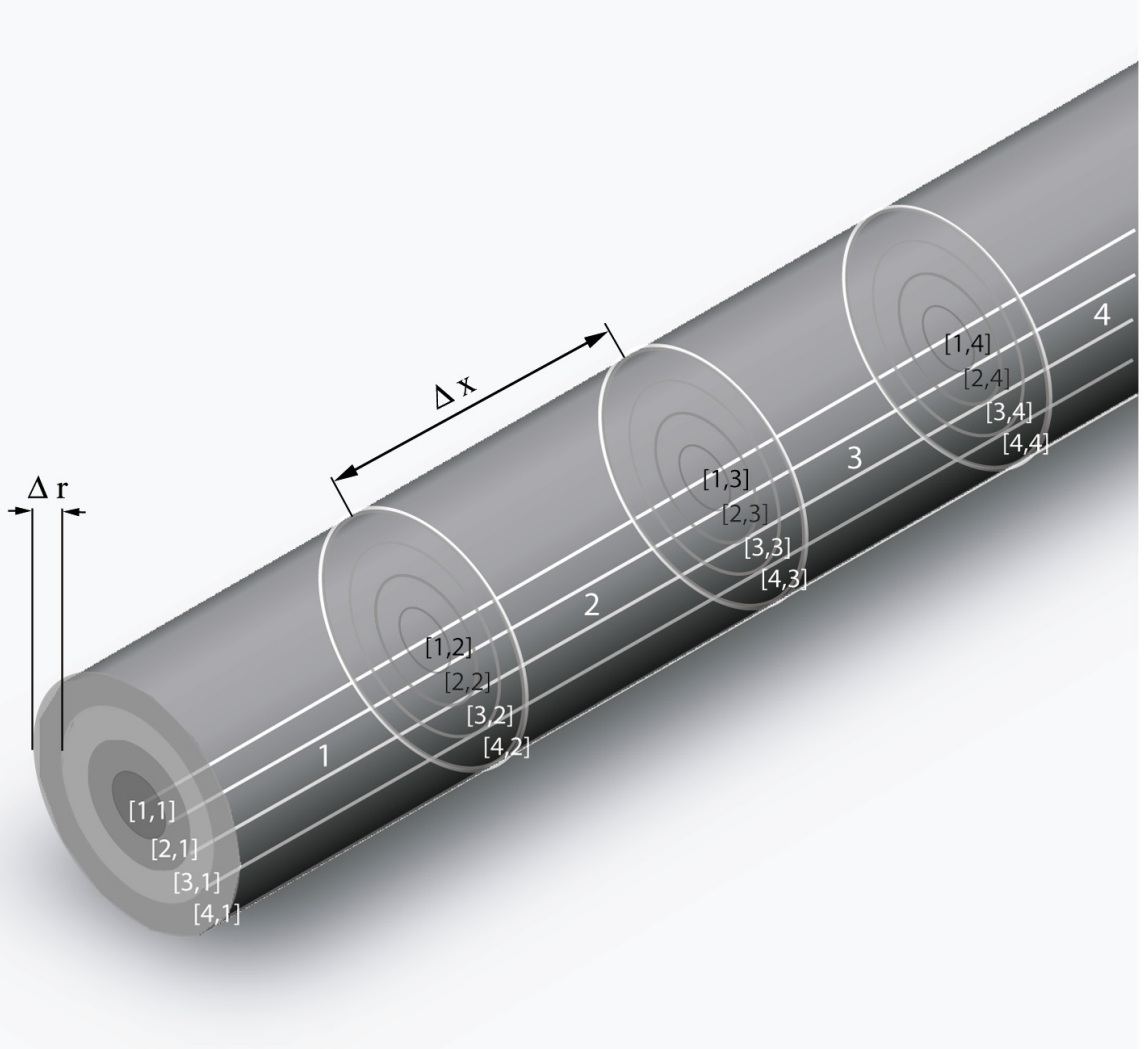
**Definition of the cell alignment within the cylindrical simulation volume. **This figure shows the geometry of the simulation volume as example with 4 × 4 cells, typical simulation volumes are set up from 100 × 100 to 500 × 500 cells. Each simulation volume is just one half of the entire volume of the implant, because its mirror symmetry allows to restrict the simulation to half the volume. The plane of mirror symmetry is on the left side of the figure. (see also Fig. 3).

**Figure 3:**
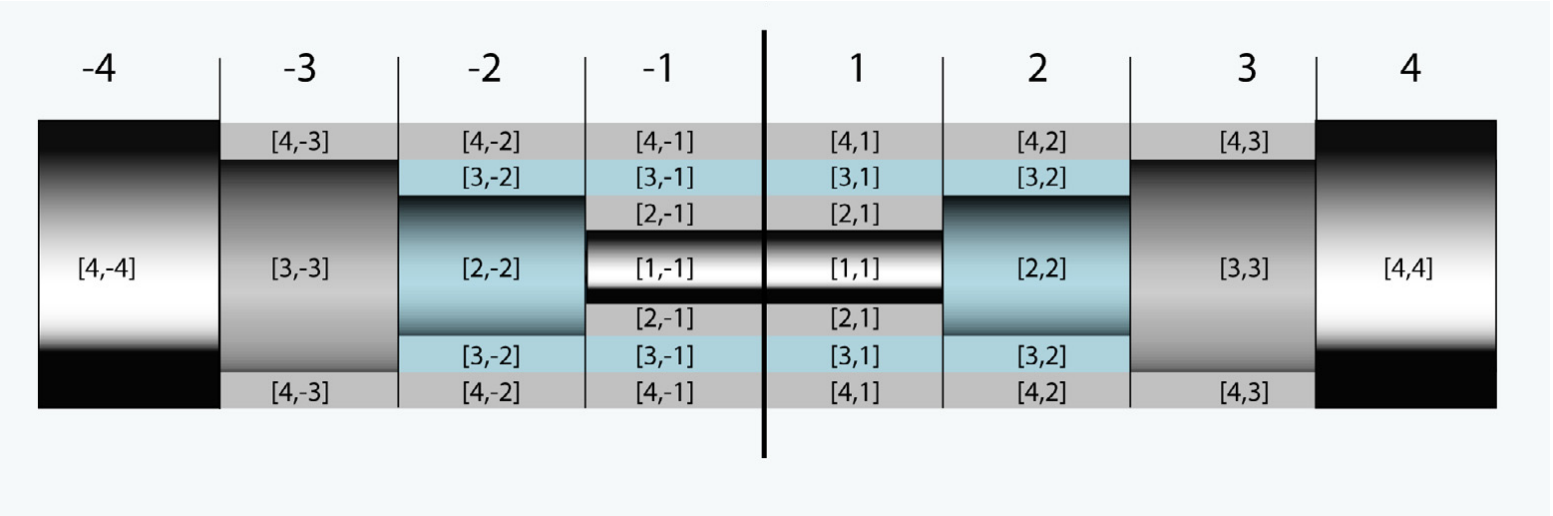
**Definition of the cell alignment within the total examined cylindrical volume for the implant. **Example of an entire volume with 8 × 4 cells. The part with complete positive indices corresponds to the simulation volume of Fig. 2. Because of the plane of mirror symmetry (dark line in the middle) only this part has to be calculated. (ΔT [r,x] = ΔT [r,-x])







The entire simulation volume is assumed as a cylinder with length *L*_sim _and radius *R*_sim_. Adequate for such a volume are cylinder coordinates. Because of the rotational symmetry, the temperature distribution is only dependent on the axial position x and the distance r from the cylinder axis (Figures 2, 3). Because of the mirror symmetry to the plane orthogonal to the cylinder axis at center, the simulation is designed to calculate the temperature distribution only on one side of the center plane. (Figures 2, 3). Using both symmetries one can divide the entire cylindrical simulation volume with length *L*_sim _and radius *R*_sim _into the following cells, which are sufficient to obtain complete information of the entire simulation volume. The calculation volume consists of n cylindrical shaped pieces with length Δ*x *and index *x *= 1, 2, ...., n denoting the position on the cylinder axis. Each sub cylinder of length Δ*x *is divided in one cylinder with radius Δ*r *and index r = 1 and m-1 shells with outer radius r* Δr, thickness Δ*r *and index *r *= 2, 3, ...., m. The entire simulation volume is represented by a two dimensional array of cells *C *[*r*,*x*] (*r *= 1,2,3,....m; *x *= 1,2,....n). Most of the cells have contact to 4 adjacent cells with contact areas different from zero (Eqs (10a, 10b)). These contact areas as well as the volumes of the cells (Eq. (10c)) only vary with the index *r*.

*A*_l_[r] [m^2^] is the contact area in both cylinder axis directions (from index *x *to *x*-1 and to *x*+1) whereas *A*_r_[r] [m^2^] is the contact area in radial direction from index r to r+1. The contact area in radial direction from index *r *to *r*-1 is identical to the area *A*_r_[r-1]. A last "shell" with cells as heat (energy) sink is placed as one boundary of the simulation volume at *r *= m + 1 and *x *= n + 1. These cells always keep a constant temperature (Δ*T *= 0), even when receiving energy during one simulation step. Partly the simulations use a second heat sink representing a blood flow through the inner volume of the implant. For this second heat sink the temperature differences of all cell elements below a radius r_flow_*Δr are also kept zero independent of the energy transfer from the cell elements with higher index r. This approximation assumes that the energy applied to the inner cylinder volume with blood flow is completely transported away from the simulation volume during one time step Δt. At *r *= 1 and *x *= 1 as well as at r = m + 1 and x = n + 1 the simulated volume has its boundaries. The symmetry and the use of cylinder coordinates imply that cells with index r = 1 or x = 1 can not exchange energy with cells at a lower index. For *x *= 1 the symmetry plane defines an identical temperature at index *x *= -1, (Figure 3) with no energy exchange across the symmetry plane. For *r *= 1 no cells with lower index r exist and therefore also no energy exchange is possible.

For all heat generating cell elements (p_cell_[r,x]>0 Eq. (9)) the physical parameters *λ*, *ρ *and *c *can be set freely. For all other cell elements they are set as tissue. The presented simulations use different metal parameters for heat generating cells (Table 1). The different physical parameters of metal and tissue for different simulation cells results in discontinuities at the metal tissue interface. Assuming the heat conduction path is from the center of one finite volume to the center of a neighboring finite volume, it takes place over two different materials with only half the diffusion length for each of the materials. Because of the much lower thermal conductivity of tissue compared to that of metal it is a good approximation to use only the diffusion length through tissue.

**Table 1:** Physical constants of tissue, titanium, iron and tantalum

material	density *ρ*	specific heat c	thermal conduct. *λ*	electric conduct. *σ*
	[kg/m^3^]	[J/(kg K]	[W/(m·K)]	[S/m]

tissue	1000 [22]	3650 [23]	0.5 [24]	0.8–8.0 [22]
saline	1003	4185		1.45*
titanium	4510	523	21.9	2.56 10^6^
iron	7870	449	80.2	10.4 10^6^
tantalum	16680	140	57.5	7.63 10^6^

* The electric conductivity of physiologic saline for the calculation of the SAR was determined to be 1.45 S/m at 22°C (295 K) and a frequency of 63.5 MHz. (Personal communication with Mr. Alexander Walkov at "Physikalisch-Technische Bundesanstalt" in Berlin, Germany)

The simulation starts with a temperature field *T *[*r*,*x*] = 0 for all *r *and *x*. For each iteration step with duration Δ*t *the total energy change for each cell element is calculated according to Eq. (9a). This change Δ*E*_tot_[*r*,*x*] of one cell is the sum of five different energies. One is due to the power loss p_cell _= *p*[*r*,*x*] defined for a cell element at index r and x. The four others are energy exchanges (Eq. (8), depending on the contact areas (Eq. (10a, b)) and the temperature differences between adjacent cells with respect to the equivalent heat diffusion length through the material of the examined region. From Δ*E*_tot _[*r*,*x*] the temperature change ΔT*[*r*,*x*] is calculated according to Eq. (9b). This value is added to the prior value (*T*_new _[*r*,*x*] := *T*_old_[*r*,*x*] + ΔT*[*r*,*x*)) for each cell during the whole iteration process. The entire simulated time *t*_sim _consists of q iterations with time interval Δ*t *(*t*_sim _= q * Δ*t*).

### Analytical model for a heat generating linear wire as test of the simulation

One test of the principal correctness of the self-coded simulation is possible by a comparison of the simulation results with those of an analytical solution. An easy straightforward analytical solution is available for an endless linear wire with radius *r*_wire_, which is heated by a constant power density P* [W/m], defined as power per wire length [W/m]. After reaching the thermal equilibrium, the power associated with P* penetrates through every cylinder surface surrounding the linear power source independent of the radius *r *(with *r*>*r*_wire_). The temperature difference ΔT between the wire surface and a point at radial position R can be calculated in a homogeneous medium from the equation for heat conduction.



**Figure 4:**
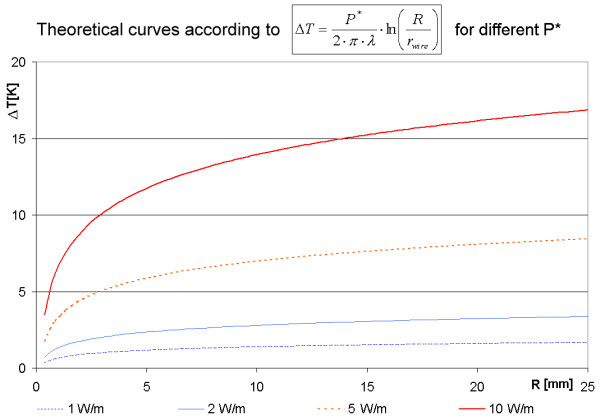
Temperature difference ΔT from wire surface with radius r_wire _= 0.05 mm to radial position R for different power densities P* [W/m]. The graphs show the temperature differences from the wire surface at position r_wire _to the position at radius R (cylinder axis defines R = 0). The starting point at r_wire _(0.05 mm) is so close to R = 0 that it coincides with R = 0 in the drawing.

The thermal capacity does not appear in Eq. (11), because of the assumed thermal equilibrium. A simulation for a wire with power loss P* and with energy exchange only in radial direction should approach, after sufficient time, similar temperature differences between the wire surface and a point at distance (R-r_wire_) (Figure 4).

### Control of simulation results

The implemented algorithm was controlled with different checks, beside the afore-mentioned comparison with an analytical model. First, the total energy uptake of the heat sink surface is added up during all iteration steps. This energy summed with the energy stored inside the simulation volume must equal the total applied energy. The energy inside the simulation volume is calculated independently from the finite volume algorithm by using the final temperature increase, the specific heat, the density and the volume of each cell.

Second, the algorithm was tested as to whether it provided similar results for identical geometries with different spatial resolution, different time resolution as well as a different size of the simulation volume surrounding the implant.

### Simulations

The cylindrical simulation volume basically is assumed as homogeneous tissue with the physical parameters (*ρ*, *λ*, c) from Table 1 and length *L*_sim _and radius *R*_sim_. A cylindrical implant with sizes *L*_imp _and *R*_imp _is placed at the center of this volume. The simulation is calculated for three different models.

1. Heat generation inside a cylinder of length *L*_imp _and radius *R*_imp_.

The heat generation is assumed to be uniform over the entire volume of *V*_imp _= *π R*^2^_imp_*L*_imp_; the entire simulation volume is assumed to be tissue.

2. Heat generation in n_r _metallic rings with rectangular cross section of *r*_wire _times *x*_wire _at radius *R*_imp_.

The rings are equally spaced over the length *L*_imp_. These power generating rings are used instead of a solenoid with *n*_r _turns to retain the cylindrical symmetry and they stand for a model where the power loss is equally distributed on and nearby the circuit paths forming the inductor. For the rings freely selectable physical parameters *c*, *λ *and *ρ *can be used (Table 1). Partly these simulations assume a flow inside the implant volume not allowing a temperature increase for the region of flow.

3. Heat generation in a cylinder shell of length *L*_imp _and thickness *r*_wire _at radius *R*_imp_.

This is an approximation of a modified solenoid structure with a high density of circuit paths on the cylinder surface. In this case, despite the assumed dense wire, most of the shell volume is tissue. Therefore the physical properties of tissue are used for the entire simulation volume.

None of the three cases exactly match the real situation. However, consistent results for all cases, which also are checked to the order of magnitude with the linear wire model of Eq. (11), could certify a realistic estimation.

## Results

### Test of Simulations: Endless wire model

**Figure 5:**
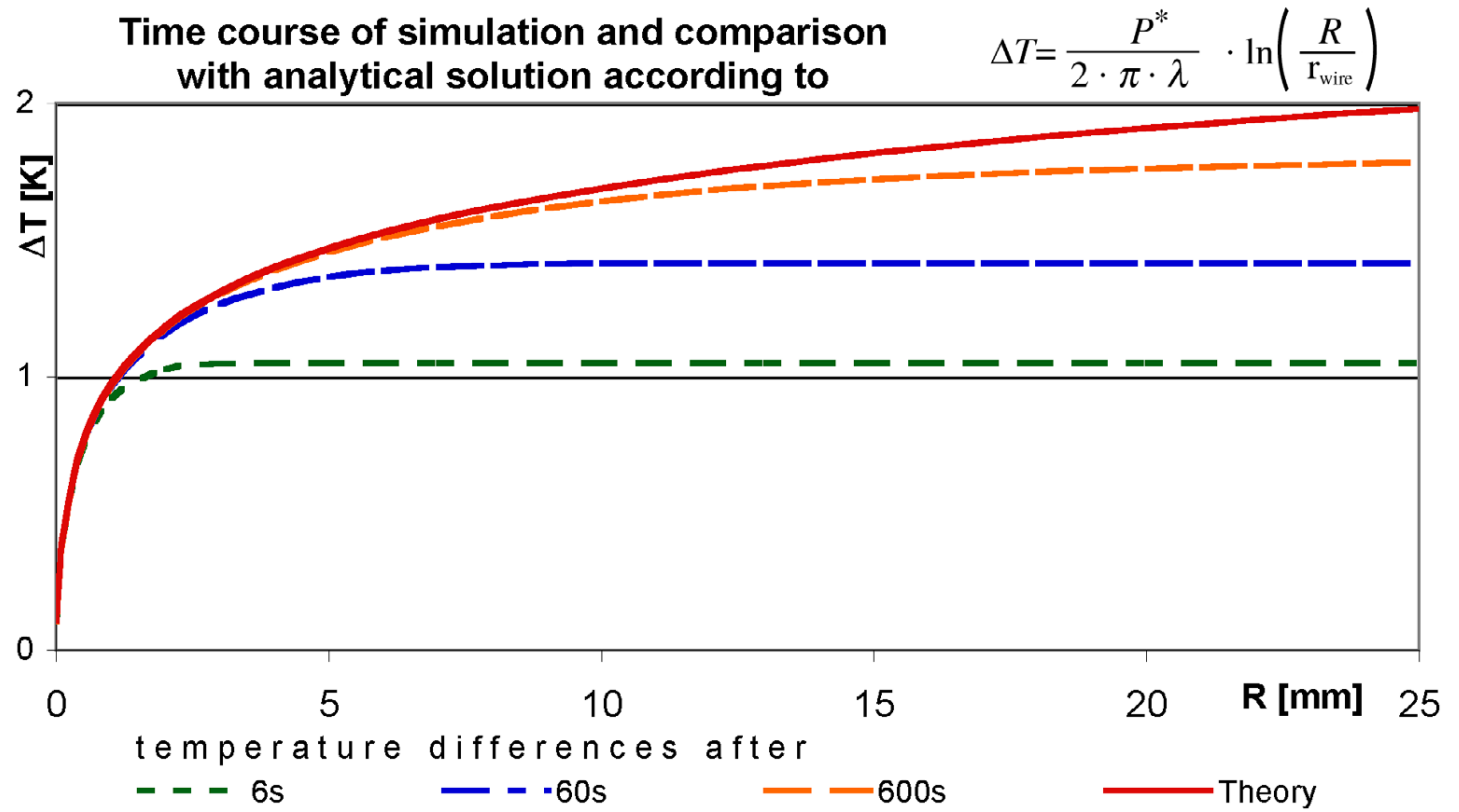
**By the simulation calculated temperature differences and comparison with the theory. **The graphs show the calculated temperature differences from the surface of a wire with radius r_wire _= 0.025 mm to a radial position R after 50 s, 200 s and 1200 s and also allows a comparison with the analytical solution of Eq. (11) (P* = 1 W/m).

For a given MRI sequence and a given resonator with known geometry it is possible to calculate the power loss density P* with Eq. (7). Using this power density P* it is possible to calculate the temperature difference between the wire surface and the difference between the wire surface and a specific radial distance after reaching the thermal equilibrium according to the model of Eq.(11). The results are summarized in Figure 5. The simulation of a linear wire is compared with a theoretical curve with respect to the same wire radius. To simulate an endless wire the energy exchange is restricted to radial direction by setting the heat sink shell temperature at maximal index x = m + 1 after each iteration not to zero but to the same value as at index x = m for all r respectively. Power generating metallic cells are assumed within the radius *r*_wire_. In radial direction, just a few cells are sufficient to describe the heat generating wire; the radial resolution Δr is chosen in such a way that n Δr - Δr/2 is equal to r_wire_. This choice takes into consideration that the center of the last cell is the reference radius r_wire _for the theoretical description according to Eq. (11). For the comparison with theory, the temperature increase in a simulation cell is set to a radial position just half between the walls of a simulation cell. During the temporal development the simulated radial temperature differences increasingly approach the analytical solution for the thermal equilibrium.

### Test of Simulations: Temporal and spatial resolution

For sufficiently short time steps Δt, the simulations show the expected behavior that the temperature increases monotonically in each simulation cell. For overly large time steps the energy flow out of a cell during a time step can become larger than the energy loss inside it. This leads to a physical incorrect situation with decreasing temperatures inside a cell. For this case the simulation produces huge deviations and the temperature differences oscillate in an unpredictable fashion during the temporal development.

**Figure 6:**
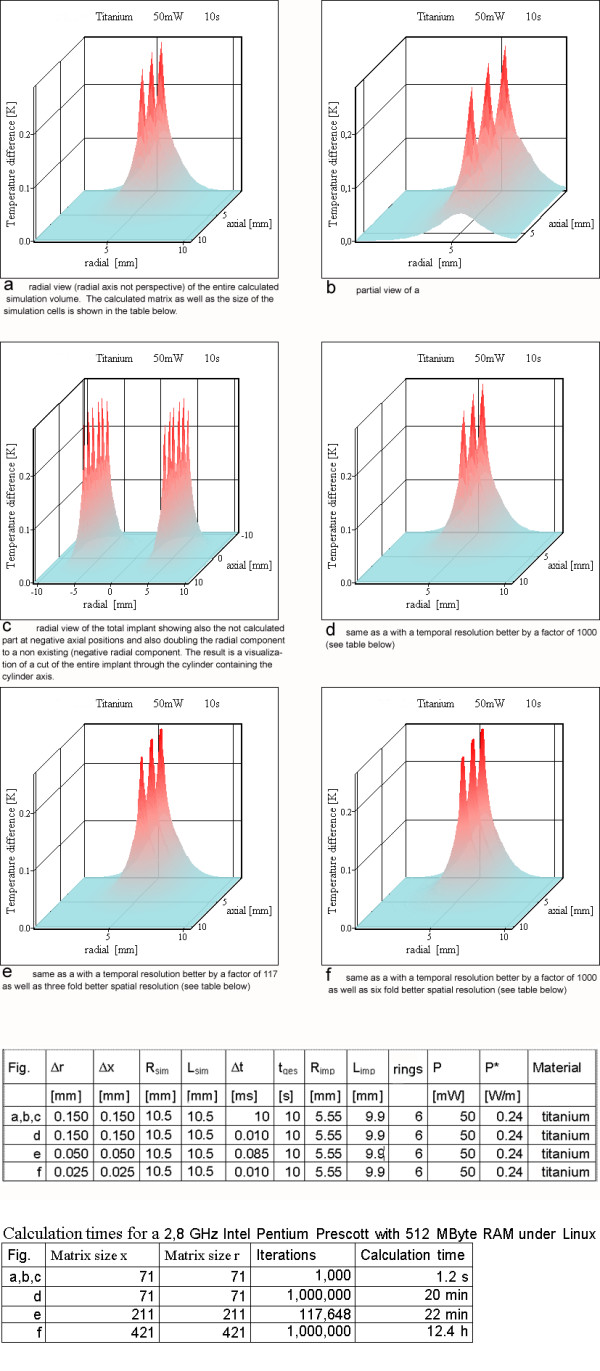
**Graphic explanation and test of the simulation with respect to temporal and spatial resolution on resonator no 2 (Table 3). **a) radial view (radial axis not perspective) of the entire calculated simulation volume. The calculated matrix as well as the size of the simulation cells and the calculation time is shown in the tables below b) partial view of a c) radial view of the total implant showing also the not calculated part at negative axial positions and also doubling the radial component to a non existing (negative) radial component. The result is a visualization of a cut of the entire implant through the cylinder containing the cylinder axis. d) same as a with a temporal resolution better by a factor of 1000 (see table below). e) same as a with a temporal resolution better by a factor of 117 as well as three fold better spatial resolution (see table below) f) same as a with a temporal resolution better by a factor of 1000 as well as six fold better spatial resolution (see table below)

Increasing the simulation volume without changing the spatial and temporal resolution as well as the size of the implant generates only slight changes (see additional file 1) at the cells corresponding to our approach that the surface of the simulation volume acts as a heat sink forcing ΔT to be zero on the surface. The tests of temporal and spatial resolution were carried out only for the short simulation period of 10 s, because the largest changes occur during the first simulation steps. Increasing solely the temporal resolution for a stable simulation affected the simulation results only minimally (Figure 6c,d). Even a huge increase by a factor of 1000 in temporal resolution between c and d does not show significant deviations. This increase allows also a comparison of Figure 6d with a better spatial resolution, which needs an increased temporal resolution.

**Figure 10:**
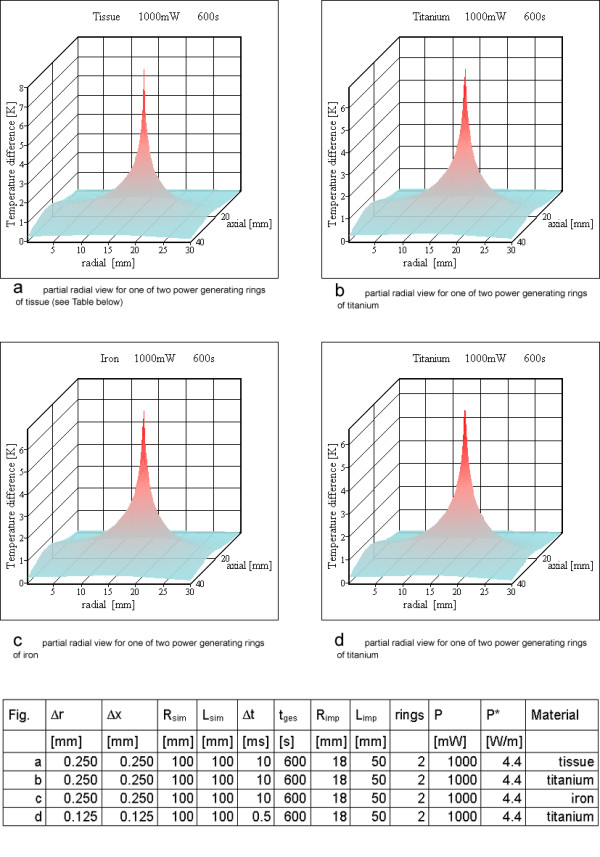
**Power loss for resonator no 1 with two rings. **a) partial radial view for one of two power generating rings of tissue (see table below) b) partial radial view for one of two power generating rings of titanium c) partial radial view for one of two power generating rings of iron d) partial radial view for one of two power generating rings of titanium

An improved spatial resolution without changes of the heat generating volume modifies the results very little (Figures 6d,e,f; 10b,d). Because an increased temporal or spatial resolution only has marginal influence on the simulation results, it is allowed to use the lowest possible temporal and spatial resolutions, which do not generate oscillatory deviations.

### Finite volume analysis

According to Eq. (7) the with *Q *normalized volumetric power loss density *P*_V _= P/(Q V_Imp_) for resonators exposed to an MRI sequence with maximum SAR (Table 2) can be calculated to 4 mW/cm^3^. Realistic vascular implants are in the order of 0.1 cm^3 ^(e.g. coronary stents; r = 1.5 mm; l = 14 mm) to 50 cm^3^, (e.g. aortic stent grafts, vena cava filters; r = 18 mm; l = 50 mm). The achievable quality factor is low in ionic surroundings, such as tissue, but increases with the insulation of the solenoid wire (unpublished diploma thesis, Karsten Behrens, University of applied sciences TFH Berlin, 2001, Department Mathematik-Physik-Chemie, Prof. Vollmann). For thin insulations, which are necessary for the retention of the mechanical properties of the vascular implants, the achievable quality factor is below 5. However, the simulation as a worst case scenario partly uses quality factors far above this value to test the safety of the implants also for these conditions. As examples, the resonators described in Table 3 were used with the physical parameters of Table 1. As simulated time, an MR sequence (Table 2) exposure of ten minutes was assumed.

**Table 2:** Data for calculation of power density *P*_V _according to Eq. (7) for an MRI sequence with an SAR of 4 W/kg (manufacturer declaration)

permeability of vacuum	*μ*_0_	[V s / (A m)]	12.6E-7
magnitude of magnetic excitation field	B_1_	[*μ*T]	25
repetition time of MRI sequence	TR	[s]	2.23
duration of one excitation	*τ*	[ms]	0.80
number of identical excitations during TR	N		246
duty cycle c_dc _= N *τ*/TR	c_dc_		0.09
pulse waveform modulation factor c_pwm_	c_pwm_		0.45
Larmor frequency = resonance frequency of LC circuit	*ν*_0_	[MHz]	63.8
power loss density P_V _= P/(Q V_imp_) (Eq. (7)	P_V_	[mW/(cm^3^Q)]	4.0

**Table 3:** Geometrical data and power losses for example resonators

Resonator	No		1	2	3
Radius of ring resonator	R_imp_	[mm]	18	5.55	1.5
Length of ring resonator	L_imp_	[mm]	50	9.9	14
Dimension of ring wire axial	x_wire_	[mm]	0.25	0.15	0.10
Dimension of ring wire radial	r_wire_	[mm]	0.25	0.15	0.10
Number of rings (turns)	n_r_		2	6	12
Volume of implant	V_imp_	[cm^3^]	50	1	0.1
Quality factor	Q		5	12.5	80
Total power loss of resonator	P_loss_	[mW]	1000	50	32
SAR inside resonator (Eq. (3b))	SAR_internal_	[W/kg]	1.8	1.7	5.1
Power loss of resonator due to SAR	P_SAR_	[mW]	0.09	0.0016	0.0005

Power losses P_SAR _and P_loss _are calculated for the MR sequence of Table 2. SAR_internal _is evaluated with Eq. (3b). P_SAR _can be calculated using SAR_internal _and the mass inside the inductor (m_imp _= *ρ*_tissue _V_imp_), whereas P_loss _is calculated with the appropriate constants according to Eq. (7). P_loss _for resonator no 2 was verified experimentally with temperature increase measurements after placing the resonator inside a phantom with known heat capacity.

### Heat generation inside a cylinder

**Figure 7:**
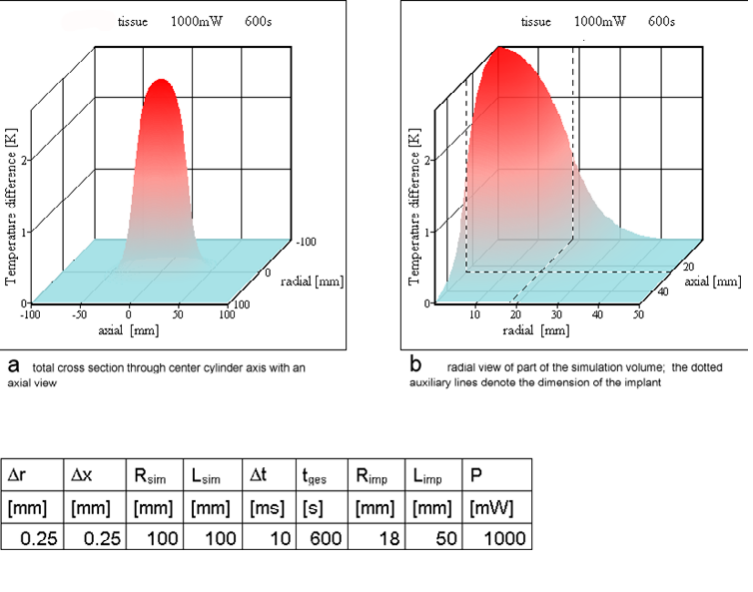
**Homogeneous power loss inside resonator no 1. **a) total cross section through cylinder containing the cylinder axis with an axial view b) radial view of part of the simulation volume; the dotted auxiliary lines denote the dimension of the implant

**Figure 8:**
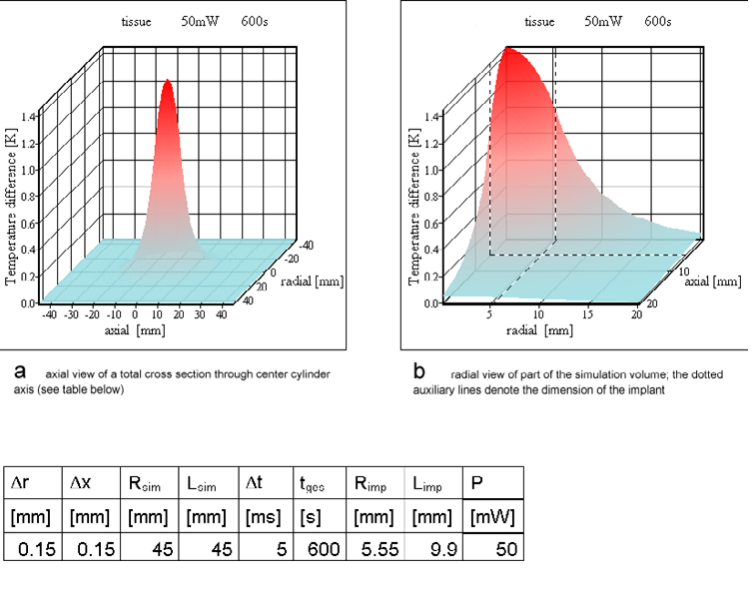
**Homogeneous power loss inside resonator no 2. **a) axial view of a total cross section through cylinder containing the cylinder axis (see table below) b) radial view of part of the simulation volume; the dotted auxiliary lines denote the dimension of the implant

**Figure 9:**
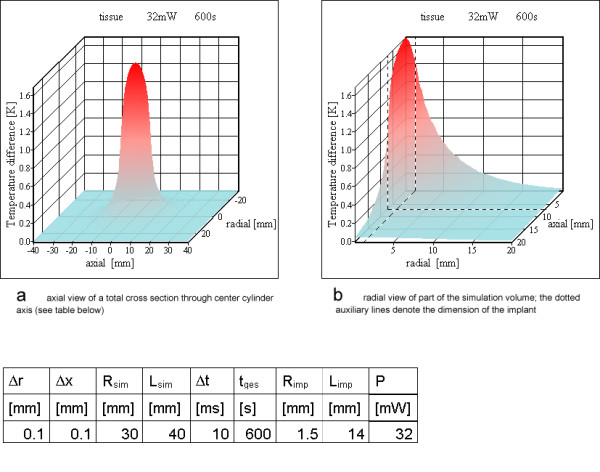
**Homogeneous power loss inside the entire resonator no 3. **a) axial view of a total cross section through cylinder containing the cylinder axis (see table below) b) radial view of part of the simulation volume; the dotted auxiliary lines denote the dimension of the implant

For the three differently shaped prototype examples (Table 3) the heat generation was assumed to be uniform over the whole volume of the resonator inductance. For one resonator (no 2) the power loss P_loss _was verified experimentally. Figures 7,8,9 show the results of these simulations. In no simulation the temperature increases reach a value of 5 K. Literature [17-21] on hyperthermia permits an estimation of the temperature resistance of normal animal cells and human cancer cells, depending on exposure time and temperature increase. The value chosen as physiologically critical, 5 K for 10 min to 15 min exposure time, is a worst case assumption, as various cells tolerate higher temperature increases as well as longer exposure times.

### Heat generation in n_r _metallic rings with rectangular cross section

**Figure 11:**
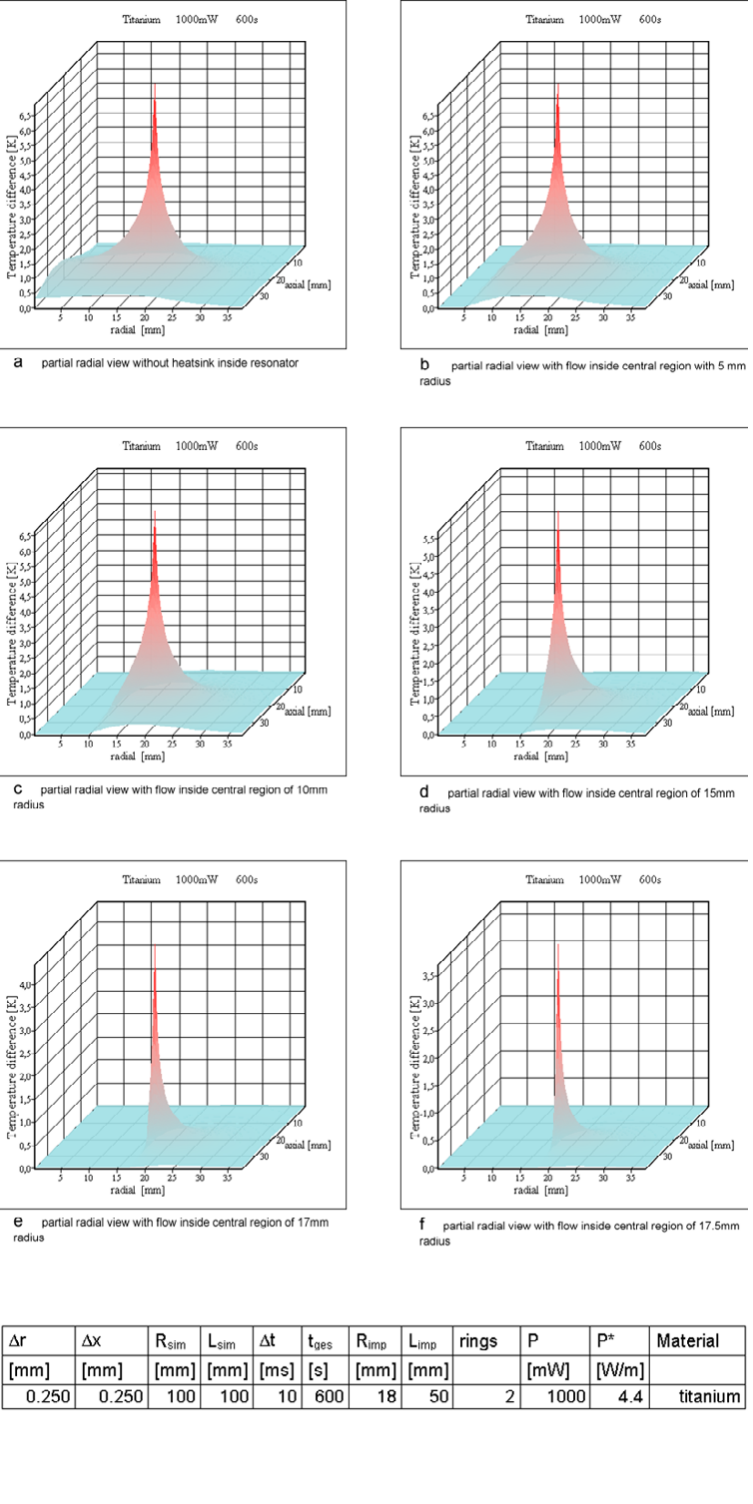
**Power loss for resonator no 1 with two rings and with simulated blood flow of varying cross sections. **a) partial radial view without heatsink inside resonator b) partial radial view with flow inside central region of 5 mm radius c) partial radial view with flow inside central region of 10 mm radius d) partial radial view with flow inside central region of 15 mm radius e) partial radial view with flow inside central region of 17 mm radius f) partial radial view with flow inside central region of 17.5 mm radius

Two rings are the worst case for resonator no 1 from Table 3 setting up a saddle coil like design. Figure 10 shows the results of the simulation. For a quality factor of 5 the temperature increases inside tissue reach 6.9 K. This is in accordance with Eq. (11). For a value of 4.4 W/m the temperature difference between the wire surface and a point at distance r = 10 cm is 9.4 K for the thermal equilibrium. Setting up a second heat sink simulating blood flow through the implant reduces the maximum temperature difference. Increasing the radius exposed to flow towards the radius of the implant increases the reduction. For an implant with the total inner volume exposed to blood flow the maximum temperature increase in the vessel wall only reaches 3.5 K (Figure 11f).

**Figure 12:**
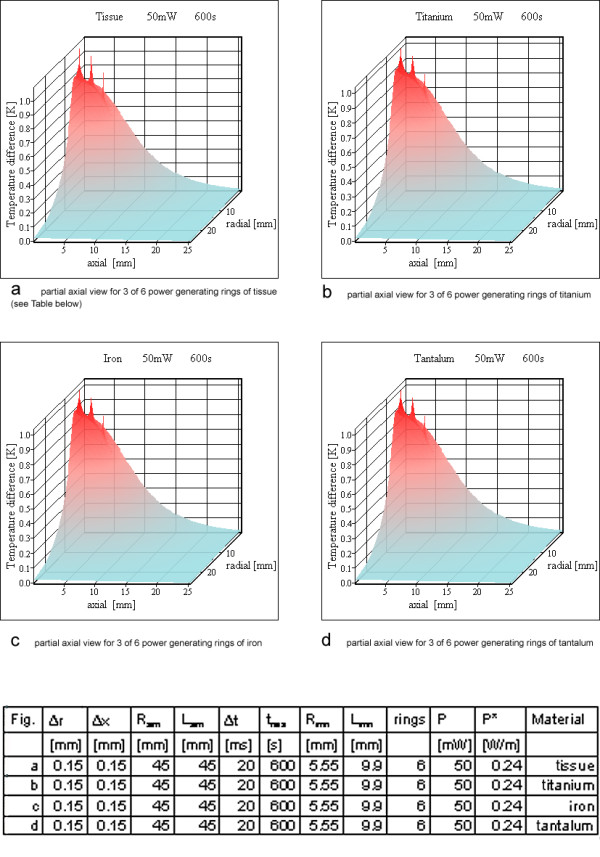
**Power loss on six rings for resonator no 2. **a) partial axial view for 3 of 6 power generating rings of tissue (see table below) b) partial axial view for 3 of 6 power generating rings of titanium c) partial axial view for 3 of 6 power generating rings of iron d) partial axial view for 3 of 6 power generating rings of tantalum

**Figure 13:**
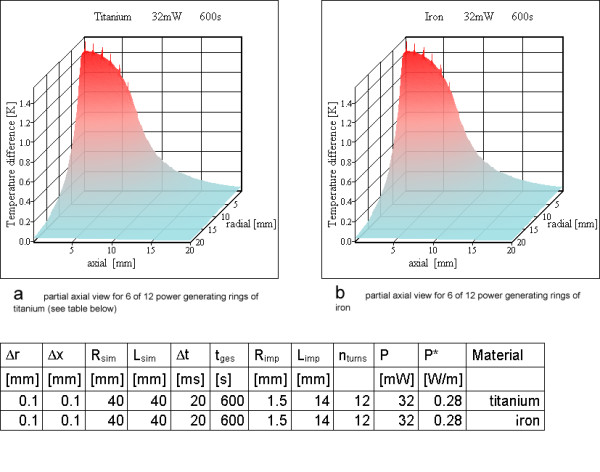
**Power loss on twelve rings for resonator no 3. **a) partial axial view for 6 of 12 power generating rings of titanium (see table below) b) partial axial view for 6 of 12 power generating rings of iron

For resonator no 2 (Table 3) 6 rings are shown in Figure 12, whereas for resonator no3 the results for 12 windings are shown in Figure 13. The material of the power generating rings has only a negligible influence on the calculated temperature maps except during the steps at the beginning of the simulation (Figures 10, 12). This is an additional proof of a correctly implemented algorithm because of two reasons. Firstly, the thermal conductivity of metal is so much larger than that of tissue, that the algorithm uses only the heat conducting path through tissue at a metal tissue interface. Secondly, the thermal capacity and the density of metal or tissue have no influence on the temperature map in the case of thermal equilibrium, because in that case heat just passes the simulation cells without changing their temperatures anymore. This argument has been mentioned already in the context of Eq. (11), which is valid for thermal equilibrium.

### Heat generation in a cylinder shell

**Figure 14:**
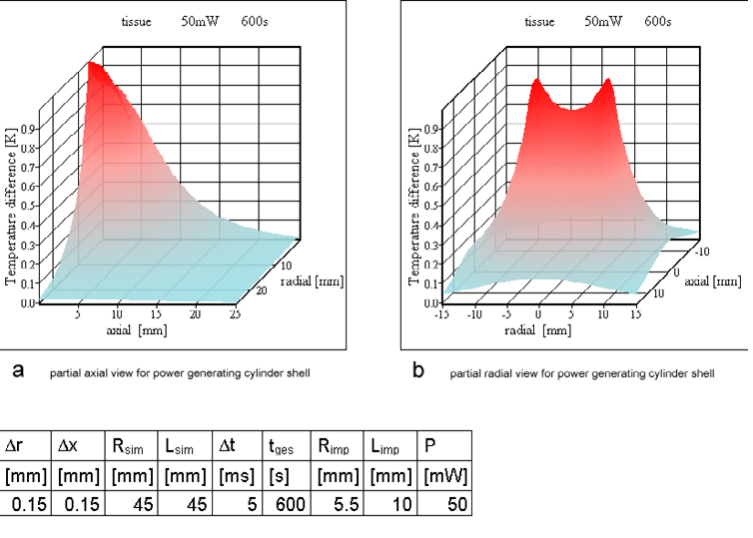
**Power loss over a cylinder shell for resonator no 2. **a) partial axial view for power generating cylinder shell b) partial radial view for power generating cylinder shell

**Figure 15:**
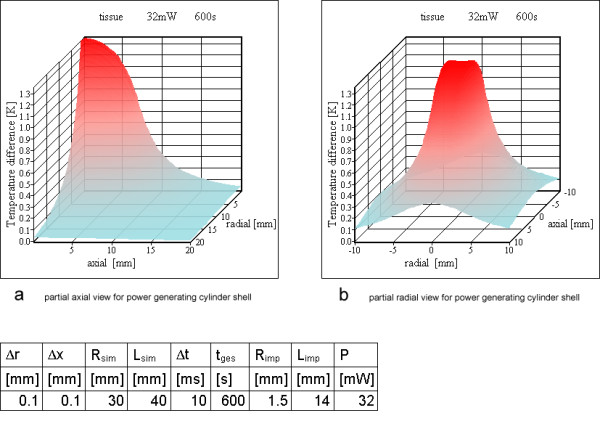
**Power loss over a cylinder shell for resonator no 3. **a) partial axial view for power generating cylinder shell b) partial radial view for power generating cylinder shell

The temperature peaks at the power generating rings become negligible for six or more rings after ten minutes simulated time. Therefore it was reasonable to choose a cylinder shell as power generating source. The simulations with a homogenous power dissipating cylinder shell instead of rings are shown from Figure 14 to 15 for resonators 2 and 3 of Table 3. None of these simulations shows a critical temperature increase.

## Discussion

### Test of simulations: endless wire model

The simulation of an endless wire uses the first of the three above mentioned models. Especially, the radius R_imp _is taken as equal to r_wire_. The condition that the wire is endless can be realized in two ways. The first way is to choose a very long L_imp _and to evaluate the temperature increases only at positions on the center plane (index x = 1) and at radial distances r small compared to L_imp_. The second way is to modify the simulation software in such a way that the energy transfer in axial direction is excluded. This can be done by considering only volume elements of the center plane (index x = 1) and setting the heat sink shell temperature (index x = 2) after each iteration not to zero, but to the same value as at index x = 1 for all r respectively.

The second way has been realized. The common and expected result was that during the temporal development the simulated radial temperature differences increasingly approach the analytical solution for the thermal equilibrium (Eq. (11)). One comparison between a simulated and the analytical result is shown in Figure 6. Although a test on agreement of both results is independent of the specific choice of the power loss density P* [W/m], we have chosen a realistic value of P* in order to obtain realistic temperature increases. The power loss density P* was calculated with Eq. (7) for the MRI sequence of Table 2 for the example resonators (Table 2) to be between 0.24 W/m and 4.4 W/m. The simulation uses value of 1 W/m.

### Test of simulations

A correct simulation of an endless wire should deliver results similar to the analytical curve of Eq. (11) using an implementation of the algorithm with no energy exchange in longitudinal (x) direction. The physical model of such a linear wire claims that the temperature difference between the wire and a cell at a certain distance increases monotonically with that distance. The theoretically calculated curve is valid after reaching the thermal equilibrium. With increasing simulated time the calculated curves show an increasingly close coincidence with the prediction of Eq.(11) (Figure 5), indicating the correctness of the algorithm.

The analytical linear wire model can also indicate which power per unit of wire length P* is safe for active magnetic implants (Figure 4). The theoretical estimation according to Eq.(11) shows for a power density larger than 2 W/m a risk of reaching temperatures increases greater than 5 K. The results do not vary with the time resolution Δ*t *as long as the value is small enough to prevent the simulation from oscillating (Figure 6c,d). Also the spatial resolution (Δ*r*, Δ*x*) does not affect the results very much as long as the temperature maps appears smooth (Figure 6d,e,f). On the other hand, the calculation time explodes with a finer resolution. An increase of the spatial resolution always requires a better temporal resolution. Especially if a metallic cell completely surrounded by tissue is divided into a few metallic cells, a superior temporal resolution is necessary. For a metallic cell completely surrounded by tissue the shortest diffusion length equals roughly half of the spatial resolution with the diffusion path through tissue. For adjacent metallic cells the diffusion length equals the spatial resolution, but the diffusion path is completely through metal with a much larger thermal conductivity. The energy transfer for an identical temperature difference through this path is at least an order of magnitude larger than the one between a metal cell and a tissue cell. This higher energy transfer requires the drastically reduced time step Δ*t *for a stable temporal development of the simulation. The calculation time for an identical simulated time increases by several orders of magnitude with metallic rings and a high spatial resolution and can easily reach computing times of several days on a normal desk top computer. Some examples for the calculation time is given in the second Table below Figure 6.

### Power loss of resonator

Achievable quality factors were derived from the construction of experimental solenoid resonator prototypes (Table 3). The quality factor for a given geometry is strongly dependent on the environment and the thickness of the wire insulation. It is reduced substantially after changing the environment from air or distilled water to saline solution. Also *Q *decreases with a thinner insulation of the wire. Using this information it is possible to conclude that the main power losses are electric losses. Losses due to eddy currents induced by the magnetic field would not change with the thickness of the electric insulation. The dominance of the electric losses is also verified by comparing the power loss from eddy currents inside the active implant (Eq. (3)) with the total power loss (Eq. (7)). For all example prototypes in Table 3, the power loss calculated with the SAR inside the resonator (Eq. (3)) is lower by several orders of magnitude than the total power loss according to Eq. (7) (Table 3).

Realistic values for the quality factor of a resonator placed in ionic surroundings such as tissue are below 5 for all example resonators of Table 3. The calculated examples are worst case assumptions as they assume the maximum achievable *Q *of 5 for resonator no 1 and therefore the maximum power loss. For resonator no 2 (Q = 12.5) and no 3 (Q = 80) the chosen value is far beyond the reachable inside an ionic surrounding. These high values for resonator 2 and 3 are chosen to pronounce the safety statement for smaller resonators with quality factor values well above achievable values. The unrealistic high quality factors do not show physiologically critical temperature increases. For resonator number 1 (with a large volume) a realistic value was chosen, because in this case the temperature increase reaches physiologically dangerous values.

Part of the simulation results could be confirmed on stents implanted inside the aorta of rabbits [9,10]. Small coated resonators with a volume below 0.5 cm^3 ^and quality factors of 3 to 4 were used. After excision and histopathologic examination, the tissue did not show any indications of heating after several MRI investigations. This is in coincidence with the simulation results, because even for small resonators with a much higher quality factor no dangerous heating was calculated.

Ten minutes of simulated time is 5 minutes less than the critical time according to the FDA regulations for imaging of the trunk assuming a SAR of 4 W/kg. But all simulations show only small changes after 10 minutes. Therefore it is unnecessary to increase the simulation time further. Nevertheless, one example of 15 minutes simulated time is given in the movie (see additional file 1) and shows only small changes in the last five minutes.

The weak influence on the distance between power generating cells and heat sink (the outer most layer) is also shown in the movie (see additional file 1). The difference between two almost identical calculations is shown. Starting from a first calculation with simulation parameters L_sim _and R_sim_, the second calculation uses doubled values of L_sim _and R_sim _without changing the spatial resolution or the other simulation parameters. This approach shifts the heat sink to a larger distance from the heat generating cell elements. As expected the heat sink layer drags down the temperature increase in its vicinity, but the effect is small if the volume is adequately chosen. The differences for identical cells between both simulations are shown by alternating both views a few times at the end of the movie (see additional file 1).

### Finite volume analysis

For all three example resonators (Table 3) the first simulation model assumes a uniform heat generation over the whole volume of the inductor. Figures 7,8,9 show the results of the simulations. All temperature increases are below the critical value of 5 K. The maximum temperature is at the cylinder axis, corresponding to the center of the vessel for a vessel implant. The uniform heat generation inside homogeneous tissue is – as discussed above – not the best approximation. The ring model is a more appropriate match for realistic conditions.

Therefore resonator 1 is assumed as a solenoid with 2 turns (Figure 10). The simulation assumes these turns as metallic rings, which should be similar to a solenoid for the question of temperature increases. For resonator no 2, six rings are calculated (Figure 12) and resonator 3 is simulated with twelve rings (Figure 13). Only the simulation for resonator no 1 shows temperature increases over 5 K, which are located on and directly adjacent to the power generating rings. For resonator 2 the temperature increase with 6 rings is slightly above 1 K (Figure 12). The same is true for resonator no 3 with twelve rings (Figure 13).

For 6 or 12 rings and after 600 s calculated simulation time, the peak values near the location of the power generating rings inside the tissue become more and more negligible. Therefore a model using a power generating cylinder shell is appropriate for resonators with a reasonable number of turns.

For resonators no 2 and no 3, the cylinder shell simulation did not show any unsafe heating and is similar to the ring calculations. Neglecting the peaks of the ring simulation the maximum temperature increase is nearly identical.

Only the simulation for resonator no 1 with two rings reaches a critical temperature value above 5 K, all others stay below this value. For a large number of rings or, stated more precisely, a high wire density at the cylinder surface, it is possible to use the cylinder shell model (compare Figures 12, 14 and Figures 13, 15). As expected, the rings cause higher maximum temperature increases according to the higher local power density. The difference between the shell generating the entire power (Figures 14, 15) and a uniform power generating volume (Figures 8, 9) is mainly a different temperature distribution inside the inductor. The former simulation first shows a moderate increase in radial direction up to the power generating shell inside the volume, followed by a decrease. The latter has a larger peak value at the axis of the cylinder and falls off continuously with greater distance from the axis. The ring model is the most preferable because of the dominance of the electric losses on and near the wire of the inductor. Using different metals without altering the other simulation parameters changes the results only marginally (Figures 10b,c; 11 b-d; 12 a, b).

The simulation worked on a "worst case" basis, neglecting in most cases all cooling effects except the energy transport due to the thermal conductivity. Especially for vascular implants in the "normal case", some blood flow and blood perfusion, causing a much faster energy transport, will reduce the temperature increases. The effect of blood flow is simulated for the example of resonator 1 with the two rings. This was the only simulation reaching physiologically critical temperature increases. Blood flow is simulated by keeping the temperature difference at zero for a central part of the cylindrical implant. Changing the dimension of the central part can simulate an implant, which may be more or less infiltrated to the vessel wall or covered by tissue (thrombosis, calcification or intimal hyperplasia). The reduced temperature increase for resonator no 1 and for various sizes of the cooling blood flow is shown in Figure 11. For an implant placed directly inside the wall, with none or only a small amount of tissue between wire and blood flow the temperature increases drop to a physiologically tolerable value (Figure 11). Nevertheless for implants with a tissue coverage of a few mm, which can be plaque or a thrombosis, critical temperatures can be reached.

With respect to the overall power absorption greater than 100 Watts inside the human body during MRI investigations with a maximum SAR, one additional Watt inside the entire body is negligible.

## Conclusion

This investigation assumes a "worst-case scenario" in different ways. Firstly, the resonator is assumed being perfectly aligned within the plane of the excitation field B_1_. Secondly, to some extent no blood flow inside the inductor (vessel) of the implant is included. Thirdly, no blood perfusion inside the tissue around the resonator is taken into account. Lastly, too large quality factors are used.

For most "normal" cases, intact active implants will therefore be less critical, but it is not possible to exclude the worst case conditions. Pathologies (thrombosis, intimal hyperplasia, plaque) may alter blood flow and perfusion in the area directly adjacent to the current paths of the resonator and certainly the resonator can be perfectly aligned to the plane of the exciting rf-field B_1_.

Especially for resonators with a large volume (such as resonator no 1) and with a small number of rings it is possible to reach critical temperature increases above 5 K (Figures 10, 11). For peripheral and especially cardiac vessels, even with an unrealistically high quality factor, the power loss is too low for dangerous heating. On the other hand, stent grafts or vena cava filters built as active implants can reach volumes of a few ten cm^3^, which may be dangerous, if the tissue around the wires of the implant is not exposed to blood flow or sufficient blood perfusion. To reduce the risk for such active implants, the quality factor has to be low. This reduces also the amplification of the MR signal the resonator is made for and can thus be dispensed with altogether.

As the above simulations assume properly working resonance circuits without any failures on the electric paths of the system, one worst case scenario was not presented. Defects such as ruptures or partial ruptures may generate a relatively high resistance over very short distances. The current flow through this resistance can produce a large power loss inside an extremely small volume. This can generate a very high power density which, even for small implants, may induce physiologically critical temperature increases for a small volume. In fact the analysis of these "hot spots" is important, because ruptures of stent struts are likely and a high power density can occur also for smaller implants. An additional investigation estimating the maximum possible power loss inside such "hot spots" and the resulting temperature maps around them is necessary to check the safety of active implants under these circumstances. This is being prepared for future publication.

The study protocols for the cited animal experiments were approved by the responsible authority (Landesamt für Arbeitsschutz, Gesundheitsschutz und technische Sicherheit, Berlin, G 0142/99).

## Authors' contributions

MB has drafted the investigation, written the manuscript, coded the simulation and contributed to the theory and part of the cited experimental work.

WV has drafted the theoretical part of the manuscript and reviewed the manuscript. Also part of the cited experimental tests for power loss, quality factor measurements and prototype constructions are from WV.

JS was responsible for the cited animal experiments and provided the in vivo images of prototypes in an animal model. He has also contributed to the medical background and has reviewed the manuscript.

DG has enabled and reviewed the manuscript, contributed to the medical background, and was helpful during the investigation with discussions.

## Supplementary Material

Additional File 1 **Movie with an example of a time developing temperature map **This movie (animated GIF) shows the time development over a period of 900 s, which is the maximum permitted time for the imaging of the trunk with an SAR of 4 W/kg (manufacturer declaration, sequence of Table 2). At the end of the simulated time, the changes in the temperature distribution become small. For complete information, a 3D axial view as well as a 3D radial view is shown simultaneously. At the end of the movie two different simulations are shown alternately, which indicate the changes of the temperature map after 900 s due to a larger simulation volume shifting the heat sink more away from cells with power loss. One of the alternating results was calculated using a 250 × 250 matrix for a distance of 0 mm to 25 mm for r and x respectively. The second map was calculated for a 500 × 500 matrix for a distance of 50 mm for r and x respectively. Only the inner 250 × 250 points are plotted for a comparable size for both calculations. It can be seen that the temperature distribution is almost identical apart from the fact that, for x≈25 mm and r≈25 mm, the simulation with more cells shows a slight deviation from zero. The simulation with the smaller matrix shows a straight zero line, which is naturally because this is the boundary condition for this simulation. The small difference points out that the boundary condition with a heat sink works very well as long as the absolute value of the gradient at the boundary is low. Click here for file
