# Supplementary figures and images for: Finite volume analysis of temperature effects induced by active MRI implants with cylindrical symmetry: 1. Properly working devices
Source: Biomed Eng Online. 2005 Apr 8;4:25. doi: 10.1186/1475-925X-4-25 (PMC1087857; doi:10.1186/1475-925X-4-25)

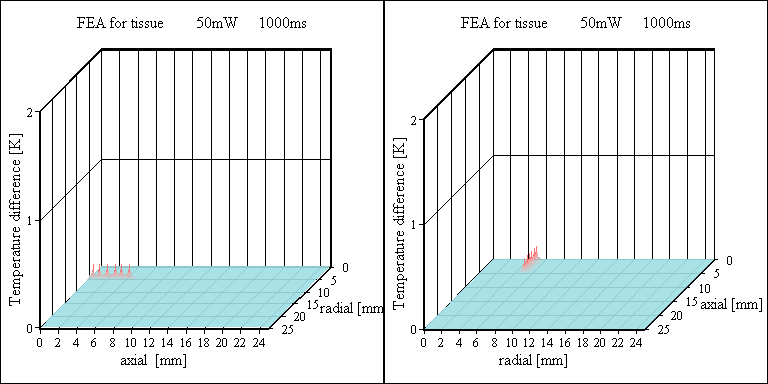

Supplement: Additional File 1 — Movie with an example of a time developing temperature map This movie (animated GIF) shows the time development over a period of 900 s, which is the maximum permitted time for the imaging of the trunk with an SAR of 4 W/kg (manufacturer declaration, sequence of Table 2). At the end of the simulated time, the changes in the temperature distribution become small. For complete information, a 3D axial view as well as a 3D radial view is shown simultaneously. At the end of the movie two different simulations are shown alternately, which indicate the changes of the temperature map after 900 s due to a larger simulation volume shifting the heat sink more away from cells with power loss. One of the alternating results was calculated using a 250 × 250 matrix for a distance of 0 mm to 25 mm for r and x respectively. The second map was calculated for a 500 × 500 matrix for a distance of 50 mm for r and x respectively. Only the inner 250 × 250 points are plotted for a comparable size for both calculations. It can be seen that the temperature distribution is almost identical apart from the fact that, for x≈25 mm and r≈25 mm, the simulation with more cells shows a slight deviation from zero. The simulation with the smaller matrix shows a straight zero line, which is naturally because this is the boundary condition for this simulation. The small difference points out that the boundary condition with a heat sink works very well as long as the absolute value of the gradient at the boundary is low. [file 1475-925X-4-25-S1.gif]
